# The potential to meet the needs of refugees and other migrants through music therapy

**DOI:** 10.1016/j.lanepe.2023.100637

**Published:** 2023-04-14

**Authors:** Viggo Krüger, Esperanza Diaz

**Affiliations:** a GAMUT – The Grieg Academy Music Therapy Research Centre, University of Bergen, NORCE Norwegian Research Centre, Norway; b Department of Global Public Health and Primary Care, University of Bergen, Norway

Music as a therapeutic medium for maintaining health and improving well-being has been described in religious and philosophical texts since ancient times. As a modern academic discipline, music therapy is approximate 70 years old. Music therapy can be described as a therapeutic individual-, group- and community-oriented approach, where music is used systematically as a medium for interaction, communication, and participation.

Today, music therapy as a planned and systematic intervention is used for a wide range of populations and across the lifespan. Because music is so important in many people's lives, it has the potential to function as a therapeutic medium and preventive tool. Music as therapy strengthens the impact of music on health and well-being. Music (therapy) may have neurological, biological, or psychological effects. Evidence for the effect of music therapy is among others described for children with disabilities,^1^ adults with mental health challenges,^2^, ^3^, ^4^ drug addiction^5^ and for elderly people with dementia.^6^ Music therapy has also a positive effect on motivation level, affective state, relational and social skills.^2^^,^^3^ Music therapy can be tailored to meet the individual needs of the child, student, patient, or health and service user.

Migration is a global challenge, and sustainable solutions to meet the needs of this population are needed from both an individual and global level. Because music therapy has proven to be important for many people and age groups, there is seminal reasons to believe that music therapy also would be beneficial for refugees and other migrants. Migration is linked to health and democratic challenges, and music and music therapy can be part of the solution, especially for some of these groups who may suffer from mental health challenges.

Young people with refugee background use music as an arena for self-expression and strengthening ethnic identity. Music therapy provides structures for sharing beliefs and hope for a better future^7^ and a safe and supportive environment for refugees and other migrants to express their emotions, build social connections, and develop coping skills.^8^ Taking part in music activities has the potential to reduce prejudice, discrimination, and aggression between groups.^9^ Music can also promote cultural understanding and create a sense of belonging. Available research in music therapy demonstrates that participation in music groups can help young refugees learn coping strategies in everyday challenges, such as attending school or doing leisure activates.^8^, ^9^, ^10^

This can be especially important for unaccompanied children who may have experienced trauma, violence during their journey to a new country.^10^ Music therapy also supports the young person in building social connections with others who have had similar experiences.^9^ Through music-making and listening, children can share their stories and connect with peers and adults in a non-verbal way.^8^ This can be important in expressing emotions and develop language skills when language creates barriers. In learning a new language, music therapy can be a helpful way to develop language skills. Through activities such as singing and playing music, children can learn new words and phrases, and practice using them in context.^8^ As such, music therapy can facilitate bridges between community approaches and treatment implementation of trauma-informed care, affording continuity and stability across situations when working with children who need to experience safety and sense of coping.^7^

Based on the foregoing, there is a need to continue exploring the link between music, music therapy and health and well-being for refugeed and other migrants. People carrying with them songs from their homeland, lullabies, or hip-hop lyrics also bring with them a very useful “first aid kit”. Music as a self-empowering technology can help people in time of crises and war. As an example, during the so called “Arab spring”, hip-hop was a driving force for many young people to let their voices be heard. To move further, we need to know more about how to people in migration can use their own resources as a mean to create resilience and empowerment. What are the mechanisms of change? What are the cultural contextual factors that provide the necessary resources for the use of music? How can be build bridges between individuals everyday life uses of music and music therapy as treatment for those in need for acute help and support? Could processes of co-creation where young people's voices are being heard through music be part of the solution?

## Contributors

All authors contributed to the concept and writing of the manuscript.

## Declaration of interests

The authors declare no competing interests.
